# CovalentInDB: a comprehensive database facilitating the discovery of covalent inhibitors

**DOI:** 10.1093/nar/gkaa876

**Published:** 2020-10-17

**Authors:** Hongyan Du, Junbo Gao, Gaoqi Weng, Junjie Ding, Xin Chai, Jinping Pang, Yu Kang, Dan Li, Dongsheng Cao, Tingjun Hou

**Affiliations:** Innovation Institute for Artificial Intelligence in Medicine of Zhejiang University, College of Pharmaceutical Sciences, Zhejiang University, Hangzhou 310058, Zhejiang, China; State Key Lab of CAD&CG, Zhejiang University, Hangzhou 310058, Zhejiang, China; Innovation Institute for Artificial Intelligence in Medicine of Zhejiang University, College of Pharmaceutical Sciences, Zhejiang University, Hangzhou 310058, Zhejiang, China; Innovation Institute for Artificial Intelligence in Medicine of Zhejiang University, College of Pharmaceutical Sciences, Zhejiang University, Hangzhou 310058, Zhejiang, China; Beijing Institute of Pharmaceutical Chemistry, Beijing 102205, China; Innovation Institute for Artificial Intelligence in Medicine of Zhejiang University, College of Pharmaceutical Sciences, Zhejiang University, Hangzhou 310058, Zhejiang, China; Innovation Institute for Artificial Intelligence in Medicine of Zhejiang University, College of Pharmaceutical Sciences, Zhejiang University, Hangzhou 310058, Zhejiang, China; Innovation Institute for Artificial Intelligence in Medicine of Zhejiang University, College of Pharmaceutical Sciences, Zhejiang University, Hangzhou 310058, Zhejiang, China; Innovation Institute for Artificial Intelligence in Medicine of Zhejiang University, College of Pharmaceutical Sciences, Zhejiang University, Hangzhou 310058, Zhejiang, China; Xiangya School of Pharmaceutical Sciences, Central South University, Changsha 410004, Hunan, China; Innovation Institute for Artificial Intelligence in Medicine of Zhejiang University, College of Pharmaceutical Sciences, Zhejiang University, Hangzhou 310058, Zhejiang, China; State Key Lab of CAD&CG, Zhejiang University, Hangzhou 310058, Zhejiang, China

## Abstract

Inhibitors that form covalent bonds with their targets have traditionally been considered highly adventurous due to their potential off-target effects and toxicity concerns. However, with the clinical validation and approval of many covalent inhibitors during the past decade, design and discovery of novel covalent inhibitors have attracted increasing attention. A large amount of scattered experimental data for covalent inhibitors have been reported, but a resource by integrating the experimental information for covalent inhibitor discovery is still lacking. In this study, we presented Covalent Inhibitor Database (CovalentInDB), the largest online database that provides the structural information and experimental data for covalent inhibitors. CovalentInDB contains 4511 covalent inhibitors (including 68 approved drugs) with 57 different reactive warheads for 280 protein targets. The crystal structures of some of the proteins bound with a covalent inhibitor are provided to visualize the protein–ligand interactions around the binding site. Each covalent inhibitor is annotated with the structure, warhead, experimental bioactivity, physicochemical properties, etc. Moreover, CovalentInDB provides the covalent reaction mechanism and the corresponding experimental verification methods for each inhibitor towards its target. High-quality datasets are downloadable for users to evaluate and develop computational methods for covalent drug design. CovalentInDB is freely accessible at http://cadd.zju.edu.cn/cidb/.

## Graphical Abstract

**Graphical Abstract:**
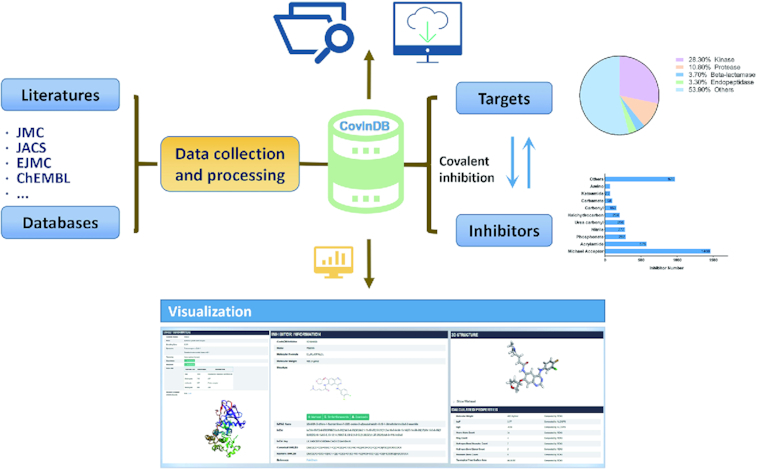
The information for covalent inhibitors and related targets is deposited in the CovalentInDB database for users to browse, retrieve and download.

## INTRODUCTION

Covalent inhibitors are usually small molecules that attach to enzymes through covalent bonds and inactive them temporarily or permanently. Typically, a covalent inhibitor contains an electrophilic functional group, which is commonly referred to as the ‘warhead’ (1) and can attack and form a reversible or irreversible covalent bond with a nucleophilic residue in the targeted protein (2). Because of the stronger covalent bonds, covalent drugs possess several advantages compared with traditional non-covalent drugs, such as higher potency, longer residence time and decreased drug resistance rate (3,4). However, covalent inhibitors have traditionally been considered to be problematic due to their potential off-target effects and toxicity issues (5), which makes pharmaceutical industry avoid to spend too much in this field for a long time. Fortunately, over the last decade 14 covalent drugs have been approved by the U.S Food and Drug Administration (FDA), which makes researchers believe that the safety issues of covalent drugs could be tackled by rational structure design that combines the strengths of covalent and non-covalent modes of drug action (5,6).

**Figure 1.**
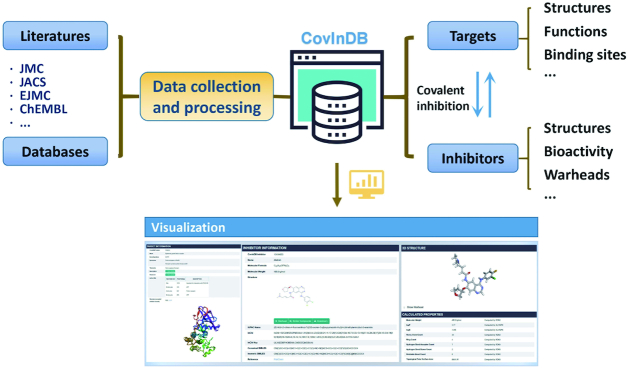
The basic framework of CovalentInDB. Data in CovalentInDB were collected from the literature and several scientific databases, and subsequently manually inspected. Various forms of information are deposited in SQLite for online visualization and downloading.

A large number of covalent inhibitors towards a wide variety of protein targets, such as kinases (7), RAS (8) and cathepsins (9), have been reported. In order to facilitate the rational design and development of covalent drugs, it is quite critical to collect and annotate the experimental data and structural information for covalent inhibitors. There are a number of public chemical biology databases, such as ChEMBL (10) and PubChem (11), providing experimental bioactivity data for small organic molecules. However, according to the data deposited in these databases, we are unable to know which ligands inhibit the target of interest through covalent mechanisms and which residues in a target are able to form a covalent bond with ligands. And this information is quite important for the study of covalent inhibitors. In 2017, Du *et al.* reported cBinderDB, the first online database for covalent binding compounds, which collected the information for 527 covalent binders and 190 related proteins (12). But unfortunately, cBinderDB is not accessible anymore. In this study, we developed Covalent Inhibitor Database (CovalentInDB), a comprehensive online resource for covalent inhibitors and related targets (Figure 1). To our knowledge, CovalentInDB is the only available and largest online database that collects the diverse information for covalent inhibitors, including the structures, bioactivity, reactive warheads, covalent reaction mechanisms and covalent-mechanism experimental verification methods for 4511 covalent inhibitors, and the binding nucleophilic residues, structures and functions of the 280 corresponding protein targets. In addition, CovalentInDB provides three data query approaches, including the text- and sequence-based search engines for proteins and the structure-based search engine for inhibitors. CovalentInDB would provide valuable guidelines to the structure optimization and warhead selection for medicinal chemists and facilitate covalent drug discovery. Moreover, some computational algorithms for covalent drug design have been developed, such as covalent docking (13), binding free energy calculation (14,15), warhead reactivity prediction (16), covalent binding site prediction (17), etc. CovalentInDB can provide high-quality data to evaluate and develop computational algorithms for covalent drug design and screening. All in all, CovalentInDB is an integrated resource that provides useful information for the mechanism study of covalent inhibition and facilitates the design of covalent inhibitors.

## MATERIALS AND METHODS

### Data collection and processing

The information in CovalentInDB was collected from scientific literatures and several scientific databases, including PubChem (11), ChEMBL (10), DrugBank (18), PDB (19) and UniProt (20).

First, the PubMed database was searched using the query keywords of ‘covalent’ and ‘irreversible’, and the publications that contain at least one of the keywords in their titles or abstracts were collected. The selected publications were manually inspected and the irrelevant publications were ruled out. The information of target proteins including protein names and species were retrieved from the collected publications. It should be noted that warheads, which determine the reactivity of covalent inhibitors, play a significant role in covalent inhibition (1). Then, the warhead for each covalent inhibitor was annotated based on the description in the corresponding publication and structural analysis. In addition, the structural transformation diagram during the covalent reaction for each warhead was also plotted according to the description reported in the literature. For each ligand–target pair, the binding site and the specific residue in the target to form the covalent bond with the inhibitor were also annotated. The binding affinity, bioactivity data, target selectivity and toxicity (if available) for covalent inhibitors were collected from PubChem, ChEMBL and the collected publications. All the protein targets in CovalentInDB were annotated with gene information, synonyms, biological functions and sequences extracted from UniProt and original publications. Moreover, the co-crystal structures bound with covalent inhibitors for some proteins were also retrieved from PDB. For those proteins without co-crystallized covalent inhibitors, other forms of structures were collected to indicate the targeted residues.

The covalent inhibitors and drugs in CovalentInDB were annotated with molecular formula, molecular weight, IUPAC name, InChI and canonical SMILES. The 3D conformations of the inhibitors were generated by the Ligprep program in Schrödinger (21). The 2D and 3D sdf files for each inhibitor in CovalentInDB are downloadable. In addition, nine important physicochemical properties related to drug-likeness calculated by RDKit (22) and ALOGPS 2.1 (23) are provided for covalent inhibitors, including molecular weight, partition coefficient (logP), aqueous solubility (logS), heavy atom count, ring count, hydrogen bond acceptor count, hydrogen bond donor count, rotatable bond count and topological polar surface area (TPSA).

### Quality assessment

In order to provide an assessment criterion of the compounds in CovalentInDB, we developed a method to rank the compounds for each target according to their experimental validation methods, bioactivity and structure similarity. Generally, the covalent labeling of proteins can be validated by several experimental techniques, including X-ray crystallography, NMR, mutation validations, mass spectrometry, isotopes labeling assay, covalent binding competition assay, dialysis assay, time-dependency inhibition assay, etc. (2). Some experimental techniques, such as X-ray crystallography, NMR and mass spectrometry, can prove the covalent mechanism directly and detect the covalent binding residues on targets. But the other methods can only be used to verify irreversible covalent inhibition, and are not accurate enough to detect the nucleophilic residues. We retrieved this information from literatures and displayed it in the result table as ‘EXPERIMENT METHOD’.

Those compounds, which have been proved to inhibit their targets through covalent-mechanism by any experimental technique, were labeled with ‘Three Stars’. Then we calculated the structural similarities between the ‘Three Stars’ compounds and the other compounds based on the Morgan fingerprints reported in the same publication. If the structural similarity of a compound is above 0.65 and the bioactivity is higher than a hundredth of the activity of the ‘Three Stars’ compound, it would be labeled with ‘Two Stars’, and the remaining compounds were labeled with ‘One Star’. In some publications, the reported compounds were hypothesized to act through covalent mechanism but no any experimental validation was provided. These compounds without experimental verification would be labeled with ‘One Star’.

### Online database implementation

CovalentInDB was designed as a relational database on an SQLite server that contains 12 tables. The website was built with the Django 2.7 framework with Python 3.6. 3Dmol.js, an object-oriented and WebGL-based JavaScript library for online molecular visualization, was embedded into the website to display the 3D structures of protein-ligand complexes and inhibitors (24). For each complex, the residue ID and chain ID of the ligand were extracted manually from the PDB file, and the surrounding residues within 5 Å of the ligand were selected and visualized by 3Dmol.js to specifically display the protein–ligand interaction around the binding site. For each covalent inhibitor, the atoms of the warhead substructure were recognized by the SMARTS-based GetSubstructMatches function in RDKit (22), and then highlighted and labeled. The sequence alignment and similarity search of target proteins are powered by BLAST+ 2.2.30 (25). The molecular structure sketcher of the ChemDoodle Web Components (26) was used as the molecular editor in CovalentInDB. Two chemical structure search engines are provided for self-edited structures: similarity-based search by the Tanimoto similarity based on the Morgan fingerprints and substructure search supported by RDKit.

## RESULTS

### Data statistics in CovalentInDB

**Figure 2.**
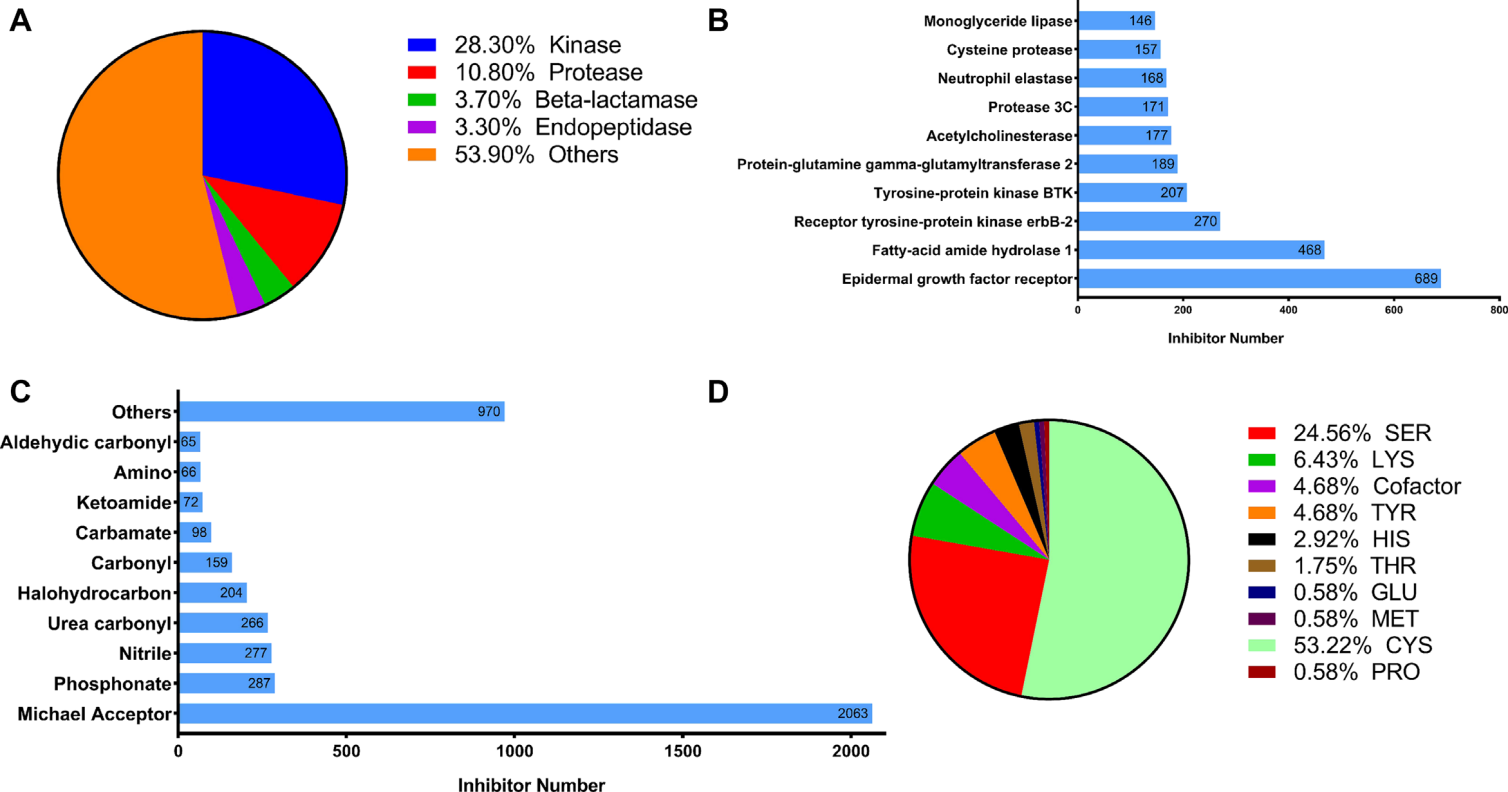
Statistics on the targeted proteins and covalent inhibitors. (**A**) The class distributions of proteins. (**B**) The top 10 targets ranked by the numbers of related covalent inhibitors. (**C**) The distributions of the electrophilic functional groups (warheads) of covalent inhibitors. (**D**) The distributions of the nucleophilic residues that form covalent bonds with inhibitors.

CovalentInDB is a comprehensive database for covalent inhibitors and their targets. It mainly contains two types of data: (i) proteins with the reactive residues and inherent covalent binding sites and (ii) small covalent inhibitors that bind to protein targets through experimentally validated covalent mechanisms. In total, the database collected the experimental data for 280 protein targets and 4511 covalent inhibitors (including 68 approved drugs) with 57 types of reactive warheads. The covalent-mechanisms of 842 compounds have been validated by experimental techniques and labeled with ‘Three Stars’. Besides, 913 compounds are labeled with ‘Two Stars’. The proteins in CovalentInDB cover 53 different species, of which 66% of the covalently modified proteins are from human and 15% from bacteria. The remaining proteins from virus, mouse, rat and pig make up 6%, 3%, 2% and 1.5%, respectively. The proteins in CovalentInDB were then classified by biological functions. We observed that the proteins in the kinase family are the most popular, and the covalent inhibitors towards kinases account for 28%, and the next is proteases which make up 29 proteins, followed by β-lactamases and endopeptidases (10 proteins for both). The covalent binding nucleophilic residues of 175 proteins in CovalentInDB were validated by experimental methods. Most targets (53%) form the covalent bonds with inhibitors through cysteine, and 25% of the targets react with covalent inhibitors through serine. Other nucleophilic residues include lysine, histidine, threonine, tyrosine and enzyme cofactor. The numbers of the covalent inhibitors for different proteins in CovalentInDB are unbalanced. Apparently, some proteins attract much more attention than the others. For example, there are 689 and 468 covalent inhibitors for epidermal growth factor receptor (EGFR) and fatty-acid amide hydrolase 1, respectively. However, the numbers of the inhibitors for those less popular proteins will decrease sharply, such as histone deacetylase and prolyl endopeptidase. As shown in Figure 2, the top 10 protein targets are epidermal growth factor receptor, fatty-acid amide hydrolase 1, receptor tyrosine-protein kinase erbB-2, tyrosine-protein kinase BTK, protein-glutamine γ-glutamyltransferase 2, acetylcholinesterase, protease 3C, neutrophil elastase, cysteine protease and monoglyceride lipase.

Similarly, the numbers of the covalent inhibitors for different warheads are also unbalanced. A total of 2063 inhibitors (45.7%) form the covalent bonds with their targets through Michael acceptor as the electrophilic functional groups, which is the most popular warhead in CovalentInDB. Phosphonate is the second most popular warhead, and it is possessed by 287 covalent inhibitors. However, some warheads are only possessed by <10 inhibitors, such as oxadiazole, hydroxamic and oxime. The top 10 warheads are Michael acceptor (2063), phosphonate (287), nitrile (277), urea carbonyl (266), halohydrocarbon (204), carbonyl (159), carbamate (98), ketoamide (72) amino (66) and aldehydic carbonyl (65). According to our statistics, the molecular weight of 67% of inhibitors is below 500, meeting the requirements of molecular weight suggested by the Lipinski’s ‘Rule of 5’ (27).

### The search engines supported by CovalentInDB

CovalentInDB provides a variety of interfaces for information retrieval and data visualization. Three data query approaches are supported by CovalentInDB: the text- and sequence-based search engines for proteins and the structure-based search engine for inhibitors. To search for a target, users can simply enter its gene name, protein name or UniProt protein identifier in the search box and press the ‘Enter’ button. The proteins that contain the keywords in their names, synonyms and encoding genes will be listed on the result page, with the query words highlighted and hyperlinks to their full information pages. Users can also use the sequence-based search engine to find any homologous enzymes of a protein of interest that have ever been modified by covalent inhibitors by pasting a FASTA format sequence in the search box. CovalentInDB will recognize the format automatically and submit the search job to the BLAST+ program, resulting in a list of similar proteins in terms of identity, alignment length, E-score and bit-score. To search for covalent inhibitors, users can use the structure-based search engine by inputting a SMILES string, uploading a MOL/SDF structure file or sketching a molecule within the ChemDoodle editor. After the query molecule has been imported, one of the three searching options (i.e., similarity, substructure or exact) can be chosen, and the high-score hits will be ranked in a datasheet along with their structures, names, formula and related protein targets.

### Information of targets and inhibitors

**Figure 3.**
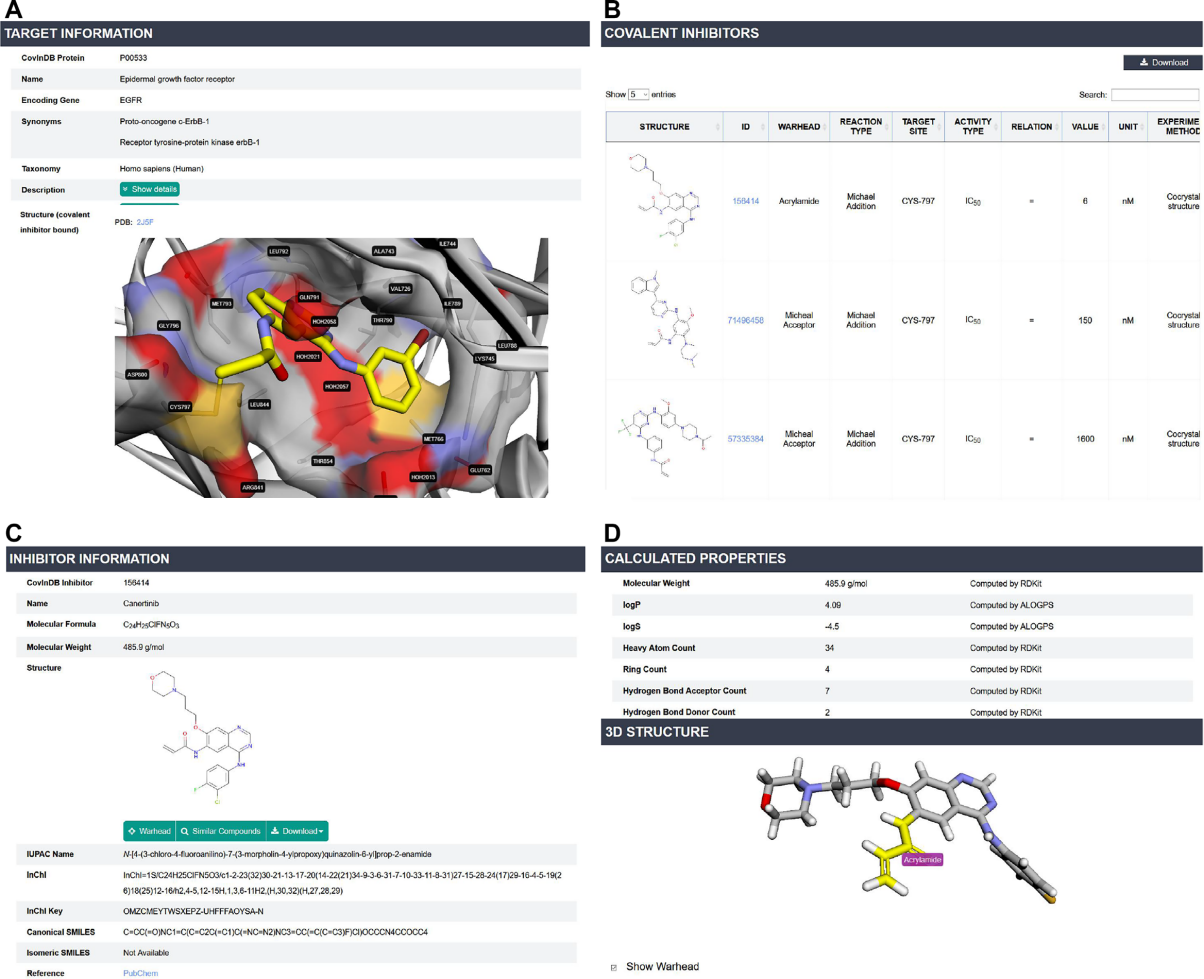
The information of targets and inhibitors in CovalentInDB. (**A**) The query results for a protein target and its co-crystal structure bound with a covalent inhibitor. (**B**) The covalent inhibitors for a protein target listed in a datasheet. (**C**) The detailed information for a covalent inhibitor. (**D**) The calculated physicochemical properties, structure and highlighted warhead substructure of the inhibitor.

The query results for proteins and covalent inhibitors are mainly organized into ‘Target Page’ and ‘Inhibitor Page’, respectively (Figure 3). In ‘Target Page’, a cocrystal structure of the protein bound with its covalent inhibitor is displayed and a molecular visualization function is embedded to display the protein-ligand interaction around the binding site, which provides valuable information to the structure-based design of targeted covalent inhibitors. The protein structure can be zoomed in to its protein–ligand binding pocket by checking ‘Site View’, and then, the residues within 5 Å around the inhibitor are shown as stick and labeled by their names and IDs. A semitransparent surface can also be added onto the whole structure or the binding pocket by checking ‘Show Surface’. For those proteins without the structures co-crystallized with covalent inhibitors, other forms of structures are displayed and can be zoomed to the targeted residues to help to understand the ligand accessibility. All the covalent inhibitors targeting this protein are listed in a table, along with their structures, IDs, rank, warheads, covalent reaction mechanism, reactive residues, bioactivity, experimental covalent-mechanism verification methods, bioassays and the hyperlinks to original publications. The hyperlink for each inhibitor will lead users to ‘Inhibitor Page’ by clicking ‘Structure’ or ‘ID’. ‘Inhibitor Page’ contains five sections: inhibitor representations, calculated properties, 3D structures, related targets, selectivity and toxicity (if available). The warhead substructure will be highlighted with a label by pressing the ‘Show Warhead’ button. CovalentInDB allows users to retrieve the similar inhibitors of a compound of interest and check their related targets, which can help users to evaluate off-target risk in the design of covalent inhibitors for a specific target. The ‘Selectivity’ section provides the bioactivity of this compounds on different targets, which is useful to evaluate its off-target risks.

### The browsing functions for quick data retrieval

**Figure 4.**
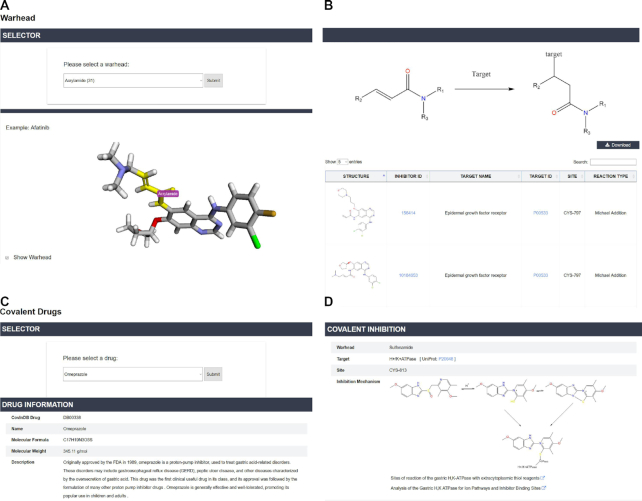
‘Warheads browse’ and ‘Covalent drugs browse’ in CovalentInDB. (**A**) Warhead selector and an example to show a warhead substructure. (**B**) The structural transformation of a warhead during the covalent reaction and covalent inhibitors with the warhead. (**C**) The detailed information for a covalent drug. (**D**) The warhead, target protein, binding site and inhibition mechanism diagram of a covalent drug.

CovalentInDB also provides four data browsing functions to retrieve the information of interest more conveniently: ‘Warheads browse’, ‘Reaction mechanism browse’, ‘Binding sites browse’ and ‘Covalent drugs browse’ (Figure 4). ‘Warheads browse’ allows users to browse the list of all covalent inhibitors possessing a specific warhead. For each warhead, a molecular visualization program is embedded to highlight the warhead structure and a diagram is plotted to show the structural transformation during covalent reaction. Similarly, ‘Reaction mechanism browse’ allows users to retrieve all covalent inhibitors that bind to their targets through the same reaction mechanism. Moreover, users can retrieve all covalent inhibitors that bind to their targets through the same residue type or co-factor by ‘Binding sites browse’. ‘Covalent drugs browse’ provides a convenient way to summarize the information for each covalent drug. By selecting a drug of interest from the 68 approved drugs and clicking the ‘Submit’ button, the detailed information for this drug will be displayed. We believe biologists and chemists can gain a better understanding of how these successful drugs achieve covalent inhibition through this information. A ‘similarity search’ function is also available for users to retrieve the similar inhibitors of this drug collected in CovalentInDB, which may be valuable for the design and optimization of covalent inhibitors.

All data in CovalentInDB are downloadable for anyone who visits our website. In ‘Download Page’, users can download the covalent inhibition information according to targets, warheads and reaction mechanisms. A compressed file that contains all data is also provided by pressing the ‘Download All’ button.

## DISCUSSION AND CONCLUSION

In the past decade, a number of covalent drugs have been approved for the treatment of various clinical diseases, which made a remarkable positive impact on human health (28). However, it is worth noting that the action modes of most covalent drugs were discovered serendipitously rather than resulting from rational design (1). With the increasing amount of experimental data for covalent inhibition studies, there is an urgent need to collect and make good use of these scattered information for rational design of covalent inhibitors. Therefore, we built CovalentInDB, a manually curated database for covalent inhibitors. The latest version of CovalentInDB contains 4511 covalent inhibitors for 280 protein targets collected by comprehensive literature searching. We believe that both wet-lab and *in silico* researchers would greatly benefit from these data. First, medicinal chemists can search a specific target and quickly get the structures and bioactivity information of its covalent inhibitors, which can guide the structural optimization and warhead selection in covalent drug design. The co-crystal structures deposited in CovalentInDB can also provide valuable information to the structure-based design of targeted covalent inhibitors for proteins of interest. Second, computer-aided drug design is playing an increasingly important role in the discovery of covalent inhibitors (29). A high-quality dataset is essential for the development and evaluation of computational algorithms for covalent drug design, such as covalent docking, warhead reactivity prediction and covalent binding free energy calculation. To our knowledge, CovalentInDB is the only available database that can meet this requirement.

We will continue to update the database to provide more extensive and accurate information of covalent inhibitors and related targets. Data in CovalentInDB will be updated every 6 months and a data uploading function will be added to CovalentInDB to allow users to upload their newly published data into the database. More experimental information of covalent inhibitors and computational tools will be added to CovalentInDB. For example, we are now collecting the crystal structures of proteins bound with covalent inhibitors in PDB. A systematic study of protein-ligand binding modes will be performed by integrating chemical genomics and structural genomics data. These data will be uploaded to CovalentInDB gradually for a better understanding of protein-ligand interactions. A covalent site identification and prediction function will be provided, and any uploaded protein can be analyzed and the residues that may be attacked by covalent inhibitors can be identified and visualized.
